# Whole genome sequencing and comparative transcriptome analysis of a novel seawater adapted, salt-resistant rice cultivar – sea rice 86

**DOI:** 10.1186/s12864-017-4037-3

**Published:** 2017-08-23

**Authors:** Risheng Chen, Yunfeng Cheng, Suying Han, Ben Van Handel, Ling Dong, Xinmin Li, Xiaoqing Xie

**Affiliations:** 1 Wuhan Oceanrice International Biotech Co.,Ltd, 30 Rongzhong International building, High-tech Development Zone, No.889 Luoyu Road, Wuhan, FL 430074 China; 2 Laboratory of Cell Biology, Research Institute of Forestry, Chinese Academy of Forestry, Beijing, People’s Republic of China; 3 Owachomo Consulting, LLC, 1101 Laveta Terrace, Ste. 19, Los Angeles, CA 90026 USA; 4 Department of Pathology and Laboratory Medicine, David Geffen School of Medicine, University of California at Los Angels, 650 Charles Young Dr, Los Angeles, CA 90095 USA

**Keywords:** Sea Rice 86, Whole genome sequencing, Transcriptome analysis, Salt resistance

## Abstract

**Background:**

Rice (*Oryza sativa*) is critical for human nutrition worldwide. Due to a growing population, cultivars that produce high yields in high salinity soil are of major importance. Here we describe the discovery and molecular characterization of a novel sea water adapted rice strain, Sea Rice 86 (SR86).

**Results:**

SR86 can produce nutritious grains when grown in high salinity soil. Compared to a salt resistant rice cultivar, Yanfen 47 (YF47), SR86 grows in environments with up to 3X the salt content, and produces grains with significantly higher nutrient content in 12 measured components, including 2.9X calcium and 20X dietary fiber. Whole genome sequencing demonstrated that SR86 is a relatively ancient *indica* subspecies, phylogenetically close to the divergence point of the major rice varietals. SR86 has 12 chromosomes with a total genome size of 373,130,791 bps, slightly smaller than other sequenced rice genomes. Via comparison with 3000 rice genomes, we identified 42,359 putative unique, high impact variants in SR86. Transcriptome analysis of SR86 grown under normal and high saline conditions identified a large number of differentially expressed and salt-induced genes. Many of those genes fall into several gene families that have established or suggested roles in salt tolerance, while others represent potentially novel mediators of salt adaptation.

**Conclusions:**

Whole genome sequencing and transcriptome analysis of SR86 has laid a foundation for further molecular characterization of several desirable traits in this novel rice cultivar. A number of candidate genes related to salt adaptation identified in this study will be valuable for further functional investigation.

**Electronic supplementary material:**

The online version of this article (doi:10.1186/s12864-017-4037-3) contains supplementary material, which is available to authorized users.

## Background

Rice (*Oryza sativa* L.) is the most important crop and a primary food source for more than half of humanity. As the world population is projected to increase to 9 billion by 2050, the world’s rice production has to increase by 25% or more to meet the demands imposed by this projected population growth. This requires identifying or breeding new rice varieties that are able to grow in marginal soils and in adverse environments. The first step toward this goal is to acquire complete knowledge of the genetic diversity in the *O. sativa* gene pool; this will enable derivation of associations between diverse genes with important agronomic traits and systematic exploitation of this rich genetic diversity [1]. Only after critical genes and alleles are identified can knowledge-based approaches be employed to integrate them into desired elite varieties using innovative breeding strategies and the most advanced targeted genome-editing technologies that allow precise and predictable gene modifications directly in established cultivars [2].

Genome-wide comparative sequence analysis is an efficient and comprehensive way to identify gene diversity among different genomes. Assisted by the rapid ascension of next generation sequencing (NGS) technology, numerous *Oryza* genomes have been sequenced since the *Oryza sativa ssp. japonica* cv. Nipponbare genome was first sequenced as the reference genome [3–5]. The genome of *O. glaberrima,* grown mainly in West Africa and evidencing traits for increased tolerance to drought, soil acidity, iron and aluminum toxicity and weed competitiveness, was sequenced recently [6]. The genomes of 3000 rice accessions collected from 89 countries were sequenced with average genome coverages and mapping rates of 94.0% and 92.5%, respectively [7]. Through whole genome sequencing-based single nucleotide polymorphism (SNP) and genome-wide association study (GWAS) analysis of 517 rice landraces, 14 agronomic traits were associated with 80 corresponding genomic sites [8]. Similarly, genome-wide association studies on 1495 elite hybrid rice varieties and their inbred parental lines associated 38 agronomic traits with 130 loci [9]. Publically accessible collections of SNPs and insertions/deletions (INDELs) identified from the sequencing data of 1479 rice accessions provide valuable resources for future association mapping studies [10].

Analysis of genes differentially expressed under various conditions can provide insights into gene function. Approximately thirty thousand expressed genes derived from *Oryza sativa* L. ssp. *japonica* cv. Nipponbare were fully sequenced and annotated as the reference transcriptome of rice [11, 12]. To obtain global views of gene activities in different tissues of *indica* and *japonica* subspecies, a high-throughput RNA-sequencing approach was applied to assess their transcriptomes through complex sequence alignment and analysis. RNA-seq identified more transcriptionally active regions and higher alternative splicing rates compared to classical, Sanger-based cDNA sequencing, and approximately thirty-eight thousand gene transcripts were identified [13, 14].

Almost 20% of the world’s cultivated lands are affected by soil salinity, which is frequently accompanied by water logging and alkalinity [15]. While most rice cultivars are susceptible to salinity, especially at their young seedling and mature reproductive stages, some rice landraces are tolerant to salinity stress through complex physiological mechanisms, including sodium exclusion, compartmentalization into the apoplasts, sequestration into older tissues, stomatal responsiveness and upregulation of antioxidants. Marker-based association mappings were conducted using salt tolerant and sensitive rice germplasms to identify polymorphic and quantitative trait loci responsible for seedling stage and reproductive stage salinity tolerance [16–21]. Microarray-based whole-genome transcript profiling of representative *indica* and *japonica* cultivars that are tolerant or sensitive to salinity stress identified potential salinity tolerant genes [22–25]. Similarly, transcriptome sequencing revealed large numbers of transcripts, including many known stress-responsive genes differentially expressed in the root and leaf, in the salinity tolerant and wild type rice varieties *Oryza coarctata* and Dongxiang, respectively, under normal or salt stress conditions [26, 27]. To identify salinity tolerance genes from a non-rice source, transcriptomes were compared between a highly salinity tolerant turf grass *Sporobolus virginicus* and rice [28].

These studies have revealed that many genes related to antioxidants, transcription factors, signal transduction, metabolic homeostasis, ion transporters and osmotic potential regulation play key roles in salinity tolerance [29]. Though salt tolerance is a complex process involving many different genes and pathways, overexpression of some individual genes involved in these biological processes enabled transgenic rice to evidence enhanced salt and drought resistance [30–35]. Submergence, often accompanied by salinity stress in coastal areas, is another major constraint to rice production. Like the existence of salt tolerant varieties, there are also highly tolerant rice cultivars that can survive up to two weeks of complete submergence. Several ethylene response factor genes were identified from the tolerant cultivars and introgressed into sensitive cultivars to promote submergence tolerance [36–38].

SR86 is a new rice cultivar domesticated from a wild strain of rice which was first found in 1986 in sea water submerged, saline-alkaline soil near the coastal region of the city Zhanjiang in Southeast China [39]. After more than 20 years of breeding and selection, SR86 retains many unique features such as the ability to grow in saline-alkaline and infertile soil, submergence and water logging tolerance and disease and pest resistance, and can grow in marginal lands while producing meaningful yields. It is considered as a strategic germplasm resource for new rice variety development due to its extraordinary salinity tolerance and acceptable average yield of ~2250 kg/ha when growing in extreme environments. Efforts are underway to either integrate high yield traits of elite rice cultivars into SR86 or to bring the salinity and submergence tolerance features of SR86 to elite cultivars to create new strains that can thrive in uncultivatable saline-alkaline lands with moderate yields. It will be of great significance to the growing world population if the estimated 950 million hectares of saline-alkaline soil worldwide can be cultivated with tolerant strains of rice [39].

In an effort to identify genes underlying the extraordinary salinity and submergence tolerance of SR86, its genome was sequenced for the first time by next generation sequencing and compared to existing rice reference genomes. Significant sequence variations and unique SNPs and INDELs were identified. Furthermore, RNA-seq-based transcriptomes of developing roots of SR86 plants grown in sea water and fresh water were compared to identify differentially expressed genes that may be involved in salinity tolerance. These results, in parallel with the genomic sequencing data, revealed a number of candidate gene families that may promote salinity tolerance.

## Results and discussion

### SR86 is salt tolerant and nutritious

SR86 grows naturally by the seaside and is highly tolerant to salt. Comparison of germination rates and salt inhibition rates (%) under different artificial saline conditions showed significant differences between SR86 and a highly salt resistant rice variety, Yanfen 47 (YF47). At a salt concentration of 0.4%, the salt inhibition rate of YF47 was 44%, while that of SR86 was only 11%. When the salt concentration was increased to 0.5%, YF47 suffered from serious saline inhibition (81%), but SR86 still had only 15% of inhibition (Table 1). Overall, SR86 had significantly higher ability to cope with high salinity measured by both germination and salt inhibition rates.

**Table 1:** Germination responses of SR86 and YF47 seeds under different artificial saline conditions

Treatment	Cultivar	Germination rate (%)^b^	Salt inhibition rate (%)^c^
Distilled water	SR86	100	0
	YF47	73	0
1/4 Hoagland solution	SR86	100	0
	YF47	80	0
0.3% NaCl^a^	SR86	87	15
	YF47	72	23
0.4% NaCl	SR86	90	11
	YF47	55	44
0.5% NaCl	SR86	87	15
	YF47	2	81
0.6% NaCl	SR86	72	30
	YF47	3	95
0.8% NaCl	SR86	2	98
	YF47	2	98
1% NaCl	SR86	0	100
	YF47	0	100
1.2% NaCl	SR86	0	100
	YF47	0	100

^a^NaCl dissolved and diluted in 1/4 Hoagland solution

^b^Percentage of germinating seeds with buds longer than half the seed size among the total of 90 seeds treated for 96 h

^c^Percentage of ((Control germination rate - treatment germination rate) /Control germination rate)

To compare the salt tolerance of these two strains under natural conditions, SR86 and YF47 seeds were germinated in sea water and local saline water conditions. As shown in Table 2, both SR86 and YF47 did not germinate well in either undiluted saline water or sea water. However, solutions of 50% local saline water or 25% sea water had almost no impact on SR86, while YF47 suffered severe inhibition under the same conditions as indicated by the less than 20% germination rate and the more than 75% salt inhibition rate.

**Table 2:** Germination responses of SR86 and YF47 seeds under different saline conditions

Treatment	Cultivar	Germination rate (%)^b^	Salt inhibition rate (%)^c^
Distilled water	SR86	100	0
	YF47	100	0
1/4 Hoagland solution	SR86	100	0
	YF47	100	0
Saline water^a^	SR86	17	83
	YF47	0	100
1/2 Saline water	SR86	95	5
	YF47	12	83
1/4 Saline water	SR86	98	2
	YF47	53	25
Sea water	SR86	0	100
	YF47	0	100
1/2 Sea water	SR86	37	63
	YF47	0	100
1/4 Sea water	SR86	98	2
	YF47	17	76
1/6 Sea water	SR86	95	5
	YF47	35	51
1/8 Sea water	SR86	93	7
	YF47	40	44

^a^Saline water is local water collected from a field containing high levels of alkali and salt

^b^Percentage of germinating seeds with buds longer than half the seed size among the total of 90 seeds treated for 96 h

^c^Represents ((Control germination rate-treated germination rate)/Control germination rate)*100

SR86 can grow without the need of applying any fertilizers or pesticides. The nutrients of this green grain are mostly higher than those of common rice grains, such as YF47, across 12 important nutrient elements, ranging from 1.6X higher for sodium, 2.9X higher for calcium, 3.6X higher for magnesium to 20X higher for dietary fiber. Consequently, SR86’s value as a subsistence crop is significantly higher compared with common rice.

Soil deterioration caused by salinization has affected 62 million hectares (20%) of the world’s irrigated lands. The problem continues to affect an estimated 14,000 ha each week, an area twice the size of Manhattan, posing a serious challenge to feed the rapid increasing world population. Based on these phenotypic data, SR86 represents an invaluable new crop for food production using salt contaminated lands and a unique genetic resource for plant breeding.

### Sequencing and characterization of the SR86 genome

As a new rice cultivar, no molecular work has been previously conducted on SR86. Before whole genome sequencing, karyotyping was performed to get a genome-wide snapshot of SR86’s chromosomes. These data (Additional file 1: Fig. S1) confirmed that SR86 contains 12 pairs of chromosomes, each with similar length to rice (*Oryza sativa*) and suggested at a gross genomic level that SR86 is a rice variety and the existing rice genome (*Oryza sativa japonica* Nippobare) can be used as a reference for SR86 genome assembly.

Four SR86 genomic libraries, three with ~300 bp inserts and one with ~2000 bp inserts, were made using KAPA LTP library preparation kits and sequenced using the Illumina HiSeq 2500 system. A total of 63.73 Gb of high quality sequence was generated, equivalent to approximately 170-fold rice genome coverage. The raw sequence reads were trimmed based on quality scores and GC bias. The cleaned reads were aligned to the temperate *japonica* Nipponbare reference genome using BWA software. 93.4% of the filtered reads were mapped to the rice reference genome and 3,800,137 variants were identified, of which 85% were SNPs and 3.3% were INDELs (Table 3). The number of variants on each chromosome was proportional to the chromosome length. Based on the *japonica* Nipponbare reference genome sequence and identified variants, we then generated a consensus genome assembly. The total length of the assembled SR86 genome is 373,130,791 bp, about 0.03% (114,728 bp) shorter than the reference rice genome (Table 4).

**Table 3:** Summary of sequence variants identified in SR86

Variation type	Chr1	Chr2	Chr3	Chr4	Chr5	Chr6	Chr7	Chr8	Chr9	Chr10	Chr11	Chr12
SNP	355,983	294,965	293,130	270,562	274,041	286,513	239,191	261,864	212,275	218,936	275,254	242,149
Ti/Tv	2.53	2.54	2.66	2.31	2.63	2.55	2.48	2.51	2.53	2.48	2.43	2.36
INDEL	18,270	15,194	14,895	12,741	13,111	14,261	12,475	12,831	10,956	11,113	13,305	12,228
Insertion	9356	7761	7496	6545	6698	7193	6502	6558	5649	5702	6911	6448
Deletion	8914	7433	7399	6196	6413	7068	5973	6273	5307	5411	6394	5780
Small insert	3	19	13	29	13	2	15	19	17	5	10	12
Large insert	23	84	15	11	16	79	11	37	22	52	18	139
Average insert	20	19	20	20	20	19	19	20	20	19	20	20
Small deletion	50	11	11	15	16	27	24	14	20	36	14	36
Large deletion	16	14	45	16	47	34	13	31	11	68	31	11
Average deletion	17	16	16	16	16	16	16	16	16	16	16	15
Structural variant	18	16	10	11	11	9	17	16	10	18	15	21
Structural insertion	5	5	0	3	5	2	3	6	4	4	8	10
Structural deletion	13	11	10	8	6	7	14	10	6	14	7	11
Multiple alterations	655	583	469	620	534	557	532	608	487	454	764	789
Total	424,146	349,943	347,169	318,172	320,667	336,769	282,385	307,495	248,844	257,257	322,081	285,209

**Table 4:** Comparison of chromosome size between the reference rice strain (*japonica* Nipponbare) and SR86

Chromosome	Reference bp	SR86 bp	Difference bp
Chr1	43,270,923	43,256,697	−14,226
Chr2	35,937,250	35,923,102	−14,148
Chr3	36,413,819	36,401,579	−12,240
Chr4	35,502,694	35,494,693	−8001
Chr5	29,958,434	29,946,993	−11,441
Chr6	31,248,787	31,236,168	−12,619
Chr7	29,697,621	29,691,082	−6539
Chr8	28,443,022	28,434,581	−8441
Chr9	23,012,720	23,005,342	−7378
Chr10	23,207,287	23,200,810	−6477
Chr11	29,021,106	29,013,401	−7705
Chr12	27,531,856	27,526,343	−5513
Total	373,245,519	373,130,791	−114,728

### Identification of unique and functionally important variants in SR86

To identify variants that were uniquely present in SR86, we analyzed the genome sequences of a core collection of 3000 rice accessions from 89 countries [7]. The analysis of the 3000 rice accessions is a relatively recent project and the entire processed variation data in the form of VCF (Variant Call Format) files are not available for public use. In order to obtain a complete picture of all genomic variants, a cloud-based high performance variant calling pipeline was developed, tested and implemented on Amazon Web Services (AWS). This analysis provided a high quality, high confidence set of variants for each of the 3000 rice accessions as compared to the *japonica* Nipponbare reference genome. The variants from each of the 3000 VCF files were then merged in order to be compared with the VCF from SR86. We sequentially removed the variants from SR86 that were also present in the merged variant file from 3000 rice accessions using the Bcdtools filter program. This reduced the number of variants in SR86 from 3,800,137 to 64,869 unique variants, out of which 18,203 were INDELs and 46,611 were SNPs.

In order to facilitate the identification of the most important variants that likely have direct effects on gene function in SR86, we computationally predicted the putative effects of the unique variants on gene function using the SnpEff tool. This resulted in the identification of 947 INDELs and 7223 SNPs as functionally high impact variants which are predicted to have major effects on protein structure or function, e.g. gain/loss of a start/stop codon, frameshift mutations, changes in splice donor or acceptor sequences, etc. (Additional files 2 and 3: Table S1 and Table S2).

### Molecular phylogenetic analysis

**Fig. 1:**
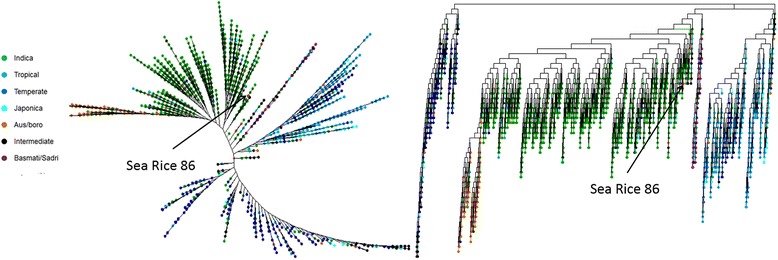
Reduced representation phylogenetic trees drawn for 3001 rice cultivars based on high quality SNPs for each strain. A total of 18,152,450 SNPs were used to create a dissimilarity matrix, which then was used to create the trees. For clarity, all branch lengths are set to one, and nearly overlapping nodes are represented as a single node. SR86 is close to the *indica* and *japonica* divergence point of the major rice varietals. *Arrows* indicate the position of SR86. Both a radial (*left*) and a horizontal (*right*) representation are shown

Based on these variant calling data, we also performed a molecular phylogenetic analysis which indicated that SR86 is a relatively ancient *indica* subspecies, phylogenetically close to the *indica* and *japonica* divergence point of the major rice varietals (Fig. 1). From this tree, we identified two Chinese rice varieties, Guang Qiu (IRGC 50320) and Bai Mi Zai 7 (IRGC 71940), that were the closest to SR86. We are now investigating if these two ancient rice varieties also have salt tolerant properties.

### Identification of SR86 chromosomal arms by INDEL-based PCR

**Fig. 2:**
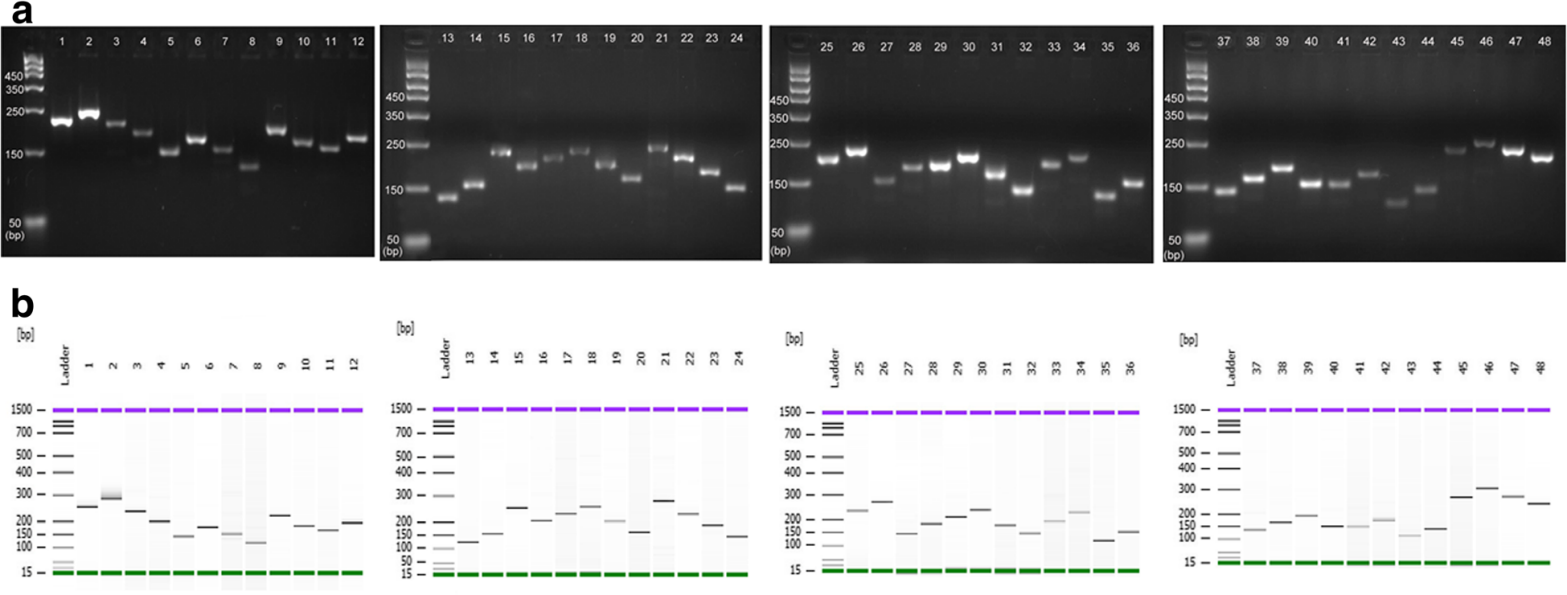
Identification of SR86 chromosome arms by INDEL-specific PCR markers. PCR products were resolved using either (**a**) agarose gel or an (**b**) Agilent 2100 Bioanalyzer. Each chromosome of reference rice (*odd number lanes*) and SR86 (*even number lanes*) was identified with a short arm-specific primer set and a long arm-specific primer set. Lanes 1–4, Chr1; lanes 5–8, Chr2; lanes 9–12, Chr3; lanes 13–16, Chr4; lanes 17–20, Chr5; lanes 21–24, Chr6; lanes 25–28, Chr7; lanes 29–32, Chr8; lanes 33–36, Chr9; lanes 37–40, Chr10; lanes 41–44, Chr11; lanes 45–48, Chr12

Given the unique features of SR86, we developed a simple method to unequivocally genotype SR86 at the resolution of each chromosomal arm. Among 18,203 unique INDEL markers, twenty-four larger than 28 bp INDELs were selected from the middle of each arm of every chromosome. The sizes of these INDELs are large enough to be visualized on an agarose gel or an Agilent 2100 Bioanalyzer after PCR amplification. By comparing the size of PCR products between the reference rice and SR86, each arm of SR86 can be easily distinguished from common rice (Fig. 2). These markers can be used collectively for SR86 identification or individually to identify a specific SR86 chromosome arm, thus providing a simple and efficient tool to assist salt tolerant breeding in the future.

### SR86 transcriptome mapping and differential gene expression

In order to identify transcripts potentially related to salt water adaptation that distinguish SR86 from non-salt tolerant strains, we designed an experiment to compare SR86 to a genomically similar but phenotypically distinct strain, R1. R1 is a hybrid *japonica indica* strain with low salt tolerance; R1 does not survive when grown in sea water. RNA-seq was performed using root RNA extracted from SR86 and R1 grown in normal fresh water (FW) for one month. Additionally, to identify transcripts that potentially enable homeostatic growth in saline conditions by SR86. SR86 was grown in sea water (SW) and FW for one month for the comparison of root expression profiles. Around 20 million 50 bp reads per sample were generated using the Illumina HiSeq 3000 sequencer, ~90% of which were mapped to Ensemble MSU6 transcript set using Bowtie2 version 2.1.0 with the default settings. The total number of SR86 transcripts expressed in root was 22,054 in FW and 22,534 in SW. The total number of R1 transcripts expressed in roots was 21,889.

**Fig. 3:**
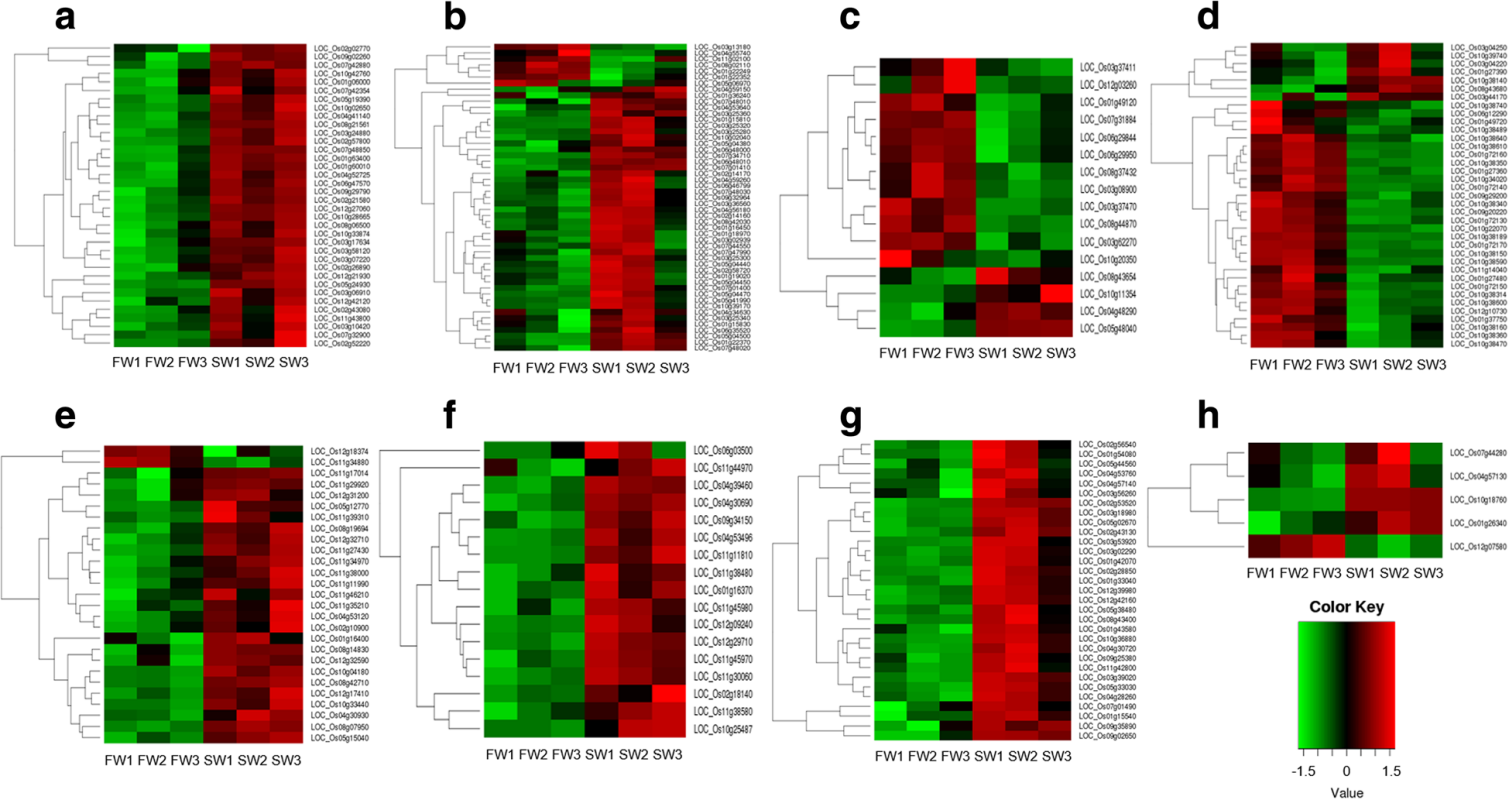
Heatmaps of all differentially expressed members in each gene family. **a** pentatricopeptide repeat (PPR) gene family, **b** peroxidase gene family, ***C*** *. mate* gene family, **d** glutathione S-transferase (GST) gene family, **e** NB-ARC domain containing gene family, **f** NBS-LRR disease resistance gene family, **g** kinesin motor domain containing gene family, **h** dirigent gene family. The scale shown applies to all heat maps; *red* depicts higher expression, while *green* represent lower

The comparison of SR86 and R1 roots grown in FW identified 3905 significantly differentially expressed transcripts (2-fold difference, FDR of 0.05; Additional files 4 and 5: Tables S3 and S4). Gene ontology (GO) analysis of the genes downregulated in SR86 vs. R1 roots using the agriGO tool [40] revealed highly significant enrichment of categories related to nucleotide metabolism (FDR = 5.2 × 10^−46^) and kinase activity (FDR = 7.0 × 10^−25^), while analysis of upregulated genes delineated fewer significant categories which were associated with basic cell functions, e.g. “membrane” and “macromolecular complex”. GO analysis of the 4560 SR86 root genes differentially expressed between SW and FW (Additional files 6 and 7: Tables S5 and S6) identified many significantly enriched categories in roots grown in SW, including those related to nucleotide binding (FDR = 2.2 × 10^−93^), response to stress (FDR = 4.2 × 10^−40^), apoptosis (FDR = 7.7 × 10^−35^), motor activity (FDR = 6.5 × 10^−18^) and peroxidase activity (FDR = 1.5 × 10^−12^). Based on the highly significant values for these categories, we further investigated the gene families in them as candidate mediators of salt adaptation (Table 5 and Additional file 8: Table S7):

1. Thirty-six members of the pentatricopeptide repeat (PPR) family, one of the largest gene families in rice with 477 members [41], were differentially expressed under saline conditions (Fig. 3a). Remarkably, all of these genes were stimulated in SR86 roots by growth in SW, whereas 28 of these genes were down-regulated in SR86 compared to R1 when both were grown in FW. Molecular evidence has predicted a role for several PPRs in coping with different biotic and abiotic stresses. A mitochondrial PPR protein, PGN, was identified to positively regulate biotic and abiotic stress responses. Arabidopsis plants with mutations in PGN displayed low resistance toward abscisic acid, glucose and high salinity [42]. A recent study identified a nucleo-cytoplasmic localized PPR protein, SOAR1 (suppressor of the ABAR-overexpressor 1) as a positive regulator of drought, salt and cold stresses [43]. These data suggest that this subset of the PPR family may represent a key component of salt adaptation by SR86.
2. Fifty-one peroxidase genes (out of 160 l Rice Genome Annotation Project) were differentially expressed between SW and FW in SR86 roots (Fig. 3b). Forty-four of those were upregulated, i.e. induced by salinity; three of these were also constitutively upregulated in SR86 compared to R1 under FW conditions (LOC_Os01g36240, LOC_Os07g47990 and LOC_Os04g53640). Increase in peroxidase activity is a common response to oxidative and abiotic stresses. It was reported that total peroxidase activity increased in response to salinity [44], which was more significant in a salt tolerant variety compared to a salt susceptible one in millet [45]. The increased peroxidase activity changed the mechanical properties of the cell wall, which in turn led to salt adaptation [44]. Over-expression of peroxidase genes in SR86 is likely to represent one of the mechanism for its salt tolerance. It is worthy of note that three genes in this group also contain high impact mutations (Additional file 8: Table S7; LOC_Os06g35520, LOC_Os01g22370 and LOC_Os01g22352 -- all predicted frameshift mutations).
3. Five dirigent genes (out of 49) were differentially expressed in SR86 roots grown in SW vs. FW (Fig. 3h); four of these were upregulated under SW growth conditions. One of these (LOC_Os10g18760) was highly expressed in SR86 roots only in SW (MMT value is >84); it was not detectable in either SR86 or R1 in FW. Dirigent proteins are involved in lignin biosynthesis through controlling phenoxy-radical coupling processes [46]. Lignin is a major component of cell walls and also Casparian strips that span the cell walls of adjacent endodermal cells to facilitate cellular control for the selective entry of water and solutes into the vascular system. Abundant expression of dirigents in SR86 roots may enhance the ability of the endodermis to respond to environmental challenges such as salt stress [47].
4. Sixteen MATE (multi-antimicrobial extrusion) protein family genes (out of 56) were differentially expressed in response to salt, 12 of which were downregulated (Fig. 3c). Seven of the suppressed genes under salt conditions were over-expressed in SR86 roots compared to R1 under FW conditions. MATE proteins are membrane transporters that can help to increase resistance to key stresses, including salinity [48]. They actively move xenobiotic substances or small organic molecules across membranes through coupling hydrolysis of ATP with import of H^+^/Na^+^ [49]. The transport direction and rate are dependent on substrate concentrations. Down regulation of these transporters can negatively regulate the influx of salt ions, suggesting a possible salt resistance mechanism. Two of the MATE genes, LOC_Os12g03260 and LOC_Os08g44870, also contain high impact mutations (Additional file 8: Table S7; one predicted gain of a stop codon and one frameshift mutation).
5. Thirty-seven glutathione S-transferases (GST) genes (out of 95) were also differentially expressed between SW and FW in SR86 roots (Fig. 3d). Different from the peroxidase genes, 31 of the 37 were downregulated, and four of these also contained frameshift mutations. Seven of the downregulated GST genes in SW were enriched in SR86 compared to R1 under FW conditions. GSTs are a ubiquitous family of multifunctional enzymes which are known to play important roles in combating different biotic and abiotic stresses via their involvement in oxidative stress metabolism and detoxification reactions [50]. By detoxifying endogenous plant toxins that accumulate as a consequence of increased oxidative stress, GSTs protect plant cells under stress conditions. Transgenic plant analysis suggested that the expression of a rice lambda class GST, OsGSTL2, in Arabidopsis provided tolerance to heavy metal and other abiotic stresses such as cold, osmotic and salt stress. Downregulation of GSTs in response to salt in SR86 is contradictory to their established roles as mediators of stress, though others have noted that individual GST genes respond differently to stress [51]. Further dissection and annotation of specific SR86 GST genes will likely yield insights into the varying responses to salt stress observed here.
6. Many members of NB-ARC and NBS-LRR gene families known to be associated with biotic stress responses were also upregulated under SW conditions: 25 of the 27 NB-ARC genes (out of 85) and all 17 of the differentially expressed NBS-LRR genes (out of 111). Although both of these gene families have been extensively studied for their roles in response to plant diseases [52], our data suggest they may also play a role in adaptation to salt stress. Indeed, De Leon et al. recently identified members of the NBS-LRR family as associated with quantitative trait loci driving resistance to salinity in Pokkali rice [53].
7. Thirty-one members of the kinesin motor domain containing gene family (out of 43) were all found to be upregulated in SR86 roots under SW conditions (Fig. 3g). These proteins encode molecular motors that are responsible for transporting cargo around the cell in association with microtubules and have been associated with such diverse functions as cell division, growth and hormone synthesis [54]. Intriguingly, there have not been many studies into their roles in stress adaptation, although changes in microtubule dynamics have been proposed as a critical component of salt stress [55]. The enrichment of kinesin motor domain family genes in SR86 under SW conditions suggests that they may represent another, novel mechanism of salt stress adaptation.

**Table 5:** Summary of the potential gene families influencing salinity adaptation

Gene family	Fraction of total members differentially expressed in SW vs. FW	Enriched in SW	Previously associated with abiotic stress adaptation
Pentatricopeptide repeat (PPR)	36/477	36 (100%)	*Arabadopsis thaliana* [42, 43]
Peroxidase	51/160	44 (86%)	*Solano lycopersicum* [44], *Setaria italica* [45]
Dirigent	5/49	4 (80%)	*Saccharum officinarum* [47]
Multi-antimicrobial extrusion (MATE)	16/56	4 (25%)	*Arabadopsis thaliana* [48]
Glutathione S-transferase (GST)	37/95	6 (16%)	*Arabadopsis thaliana* [50], *Solano lycopersicum* [51]
NB-ARC domain containing	27/85	25 (93%)	Not reported
NBS-LRR disease resistance	17/111	17 (100%)	*Oryza sativa* [53]
Kinesin motor domain containing	31/43	31 (100%)	*Arabadopsis thaliana* [55]

## Conclusions

The SR86 genome and transcriptome analyses reported here provide an invaluable new resource for further molecular studies on this unique germplasm, shed insights into the genetic basis of the similarities and differences between sea rice and common rice and help to reveal possible molecular mechanisms underlying salt tolerance. The analyses identified a distinct cohort of genes whose expression and sequence may contribute or are causative to the salt tolerance phenotype of SR86.

## Methods

### Chromosome preparation

SR86 roots were collected from Zhanjiang, Guangdong Province, China. Root tips were harvested from sprouted seeds and incubated in 2 mM 8-hydroxyquinoline at 20 °C for 2 h to promote accumulation of metaphase cells. Roots were then fixed in methanol:acetic acid (3:1) and digested in 2% cellulose and 1% pectolyase at 37 °C for 1.5 h before mounting on slides. An Olympus BX51 microscope with attached camera was used to image Giemsa-stained spreads.

### Germination experiments

Seeds from indicated cultivars were placed on filter paper in Petri dishes and doused with the indicated solutions. Petri dishes were covered and placed in growth chambers kept at 30 °C and 60% humidity; seeds were washed with indicated solutions once daily. Germination rates were calculated at day 10.

### Sample preparation, genome and transcriptome sequencing

Genomic DNA was extracted from fresh root tissue using the DNeasy Plant Mini Kit (Qiagen). Two micrograms genomic DNA was divided equally into two tubes and sheared into ~300 bp and ~2000 bp fragments separately using M220 ultrasonicator (Covaris). DNA libraries were prepared with the KAPA LTP DNA Library Prep Kit (KAPA Biosystems) according to manufacturer’s instructions, and sequenced by whole-genome paired end sequencing using an Illumina HiSeq 2500. For transcriptome sequencing, SR86 was planted in fresh water while R1 was planted in sea water and fresh water. Roots were harvested in three biological replicates one month after planting. Total RNA was extracted from roots using the RNeasy Plant Mini Kit (Qiagen). One microgram was used for mRNA library preparation with KAPA Stranded mRNA-Seq Kit (KAPA Biosystems) according to the manufacturer’s instructions. The libraries were sequenced using Illumina HiSeq 3000 platform. Raw sequence reads were mapped to Ensemble MSU6 transcript set using Bowtie2 version 2.1.0 with default setting. Gene expression levels were estimated using RSEM v1.2.15 and normalized with the TMM (trimmed mean of M-values) method. Differentially expressed genes were identified using the edgeR program. Differential expression was determined by the generalized linear model (GLM) likelihood ratio test. Genes showing altered expression with FDR < 0.05 and more than 2-fold changes were considered differentially expressed. For data quality control, FASTQC was used to check the raw fastq data quality and Trimmomatic was used to remove adaptors and to trim quality bases. After adapter clipping, we removed leading and trailing ambiguous or low quality bases (below Phred quality scores of 3). Trimmomatic works with a user-defined window spanning the read from 5′ to 3′ and removes bases only at the 3′-end; we set up a window length of 4 and a quality threshold Q of 20. When the average quality drops below 20, the 3′-end is clipped.

### Sequence alignment, mapping and variant calling

Raw sequence reads were trimmed based on quality scores and GC bias. The cleaned reads were aligned to the temperate *Oryza sativa ssp. japonica* cv. Nipponbare reference genome, Release 7 of the unified-build release Os-Nipponbare-Reference-IRGSP-1.0 (IRGSP-1.0), using BWA software. The alignment results were then merged and indexed as BAM files. Variant calling was performed on alignment file using the Genome Analysis Toolkit 3.0–2 (GATK) and Picard package V1.89. The identified variants were annotated using controlled vocabulary terms; the sequence changes and their impacts were predicted using the SnpEff method. A consensus genome build for SR86 was generated based on the *japonica* Nipponbare reference genome sequence and the identified variants.

### Molecular phylogenetic analysis

For SR86 and each of the 3000 rice genomes, we first listed high-quality and high-confidence SNPs that had a read-depth of 4 or more, mapping quality of 30 or more and quality of 50 or more; and then merged them all into one single.vcf file that defined the genotype for each SNP across all cultivars. This resulted in a file with 18,152,540 total SNPs. Next, the R-based tool, SNPrelate, was used to calculate the dissimilarity matrix between all 3001 genomes based on the presence/absence of these SNPs. Results were exported out of R and used to draw the phylogenetic tree with the tool DARWIN.

### Development of genome-wide INDEL markers

Twenty-four INDELs larger than 28 bp were selected for the development of genome-wide INDEL markers: one in the middle of the short arm (p) and one in the middle of the long arm (q) for each chromosome. The forward and reverse primers targeting these INDEL-carrying sequences were designed using the generic primer interface of BatchPrimer3 (https://probes.pw.usda.gov/batchprimer3/). The criteria were as follows: product size 100–300 bp, primer size 18–27 bp, GC content 20–80%. The sequences of the reference genome [*Oryza sativa* (rice)] and all primer pairs were BLAST searched against NCBI sequence database (https://blast.ncbi.nlm.nih.gov/Blast.cgi) to verify the PCR product in the reference.

The 24 pairs of primers were synthesized by IDT (Integrated DNA Technologies). PCRs were conducted in 25 μL reactions containing 2.5 μL 10X reaction buffer (NEB), 0.125 μl Taq (NEB), 0.5 μL of 25 mM dNTP (Amersco), 0.5 μL of 10 μM primers, 10 nanograms of SR86 and reference rice DNA and 20.375 μL nuclease-free water (NEB). PCR reactions were carried out as follows: 95 °C, 30 s; 30 cycles of (95 °C, 30 s; 52 °C, 30 s; 68 °C, 30 s) and 5 min extension at 68 °C. PCR products were analyzed with a Bioanalyzer 2100 according to the manufacturer’s instructions (Agilent).

## Additional files

Additional file 1: Karyotype of Sea Rice 86. SR86 contains 12 pairs of chromosomes, each with similar length to rice (*Oryza sativa*). A metaphase spread stained with Giemsa is depicted. (TIFF 681 kb)

Additional file 2: Predicted unique, high impact structure/function altering SNPs. (XLSX 248 kb)

Additional file 3: Predicted unique, high impact structure/function altering INDELs. (XLSX 51 kb)

Additional file 4: Differentially expressed genes upregulated in SR86 vs. R1 roots grown in fresh water and their gene ontology. (XLSX 340 kb)

Additional file 5: Differentially expressed genes downregulated in SR86 vs. R1 roots grown in fresh water and their gene ontology. (XLSX 316 kb)

Additional file 6: Differentially expressed genes upregulated in SR86 roots grown in sea water vs. fresh water and their gene ontology. (XLSX 473 kb)

Additional file 7: Differentially expressed genes downregulated in SR86 roots grown in sea water vs. fresh water and their gene ontology. (XLSX 338 kb)

Additional file 8: Differentially expressed gene families that are likely associated with salt tolerance. (XLSX 241 kb)

## Abbreviations

ATP: Adenosine triphosphate
AWS: Amazon Web Services
BLAST: Basic local alignment search tool
bp: Base pairs
BWA: Burrows-Wheeler Aligner
Chr: chromosome
FW: Fresh water
GATK: Genome Analysis Toolkit
Gb: Gigabytes
GST: Glutathione S-transferase
GWAS: Genome-wide association study
INDEL: Insertions/deletions
LRR: Leucine-rich repeat
MATE family: Multi-antimicrobial extrusion protein family
NB-ARC domain: Nucleotide-binding adaptor shared by APAF-1, R proteins, and CED-4 domain
NBS: nucleotide-binding site
NGS: next generation sequencing
PPR: Pentatricopeptide repeat
RNA-seq: RNA sequencing
SNP: Single nucleotide polymorphism
SR86: Sea Rice 86
SW: sea water
TMM: Trimmed mean of M-values
VCF files: Variant call format files
YF47: Yanfen 47
