# Supplementary figures and images for: Whole genome sequencing and comparative transcriptome analysis of a novel seawater adapted, salt-resistant rice cultivar – sea rice 86
Source: BMC Genomics. 2017 Aug 23;18:655. doi: 10.1186/s12864-017-4037-3 (PMC5569538; doi:10.1186/s12864-017-4037-3)

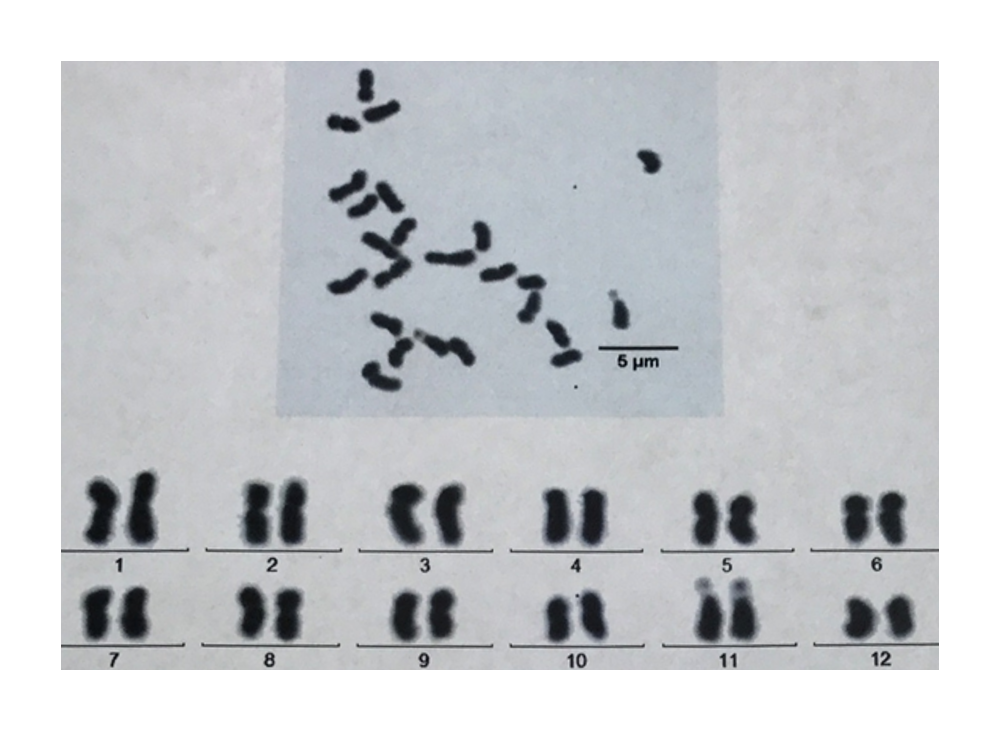

Supplement: Supplementary file 1 — Karyotype of Sea Rice 86. SR86 contains 12 pairs of chromosomes, each with similar length to rice (Oryza sativa). A metaphase spread stained with Giemsa is depicted. (TIFF 681 kb) [file 12864_2017_4037_MOESM1_ESM.tif]
